# The Relationship Between Perimesenteric Fat and Measures of Central Adiposity in Young Adults

**DOI:** 10.7759/cureus.73097

**Published:** 2024-11-05

**Authors:** Francis St-Onge, Allyson Whitsett, Jean-Felix St-Onge, Jeriel Cruz, Rajab Abdulsadek, Husein Alghurairy, Tarek Alambrouk, Haider Hilal, James Coey, Najla Yussuf Moosa

**Affiliations:** 1 School of Medicine, Northumbria University, Newcastle Upon Tyne, GBR; 2 School of Medicine, St. George's University School of Medicine, St. Georges, GRD; 3 Medicine, Hull University Teaching Hospital, Hull, GBR; 4 School of Medicine, St. George's University School of Medicine, Newcastle Upon Tyne, GBR; 5 School of Medicine, University of Sunderland, Sunderland, GBR

**Keywords:** body mass index, metabolic disorders, obesity, superior mesenteric artery, visceral adipose tissue

## Abstract

Visceral fat has been identified as a key contributor to metabolic disorders owing to its association with decreased adipocytokine function. Perivascular adipose tissue (PVAT), a specialized local deposit of adipose tissue surrounding arteries, has been shown to regulate vascular tone through adipocytokine functions but is compromised in obesity, contributing to increased vascular resistance. This study aimed to investigate the correlation between PVAT of the superior mesenteric artery (SMA), visceral adipose tissue (VAT), body mass index (BMI), and waist-to-hip ratio (WHR). WHR and BMI were anthropometric measurements used to assess body composition. A GE LOGIQ ultrasound system with a 12 MHz abdominal probe transducer was used to measure VAT at the SMA, VAT at the umbilicus, and PVAT of SMA in 31 healthy participants, male and female, aged 18-30. Pearson’s correlation matrix was generated to assess the strength of the correlation between variables, and ANOVA was used to assess the statistical significance of Pearson’s correlation coefficient (r) values. PVAT positively correlated with BMI, WHR, and VAT (p<0.05). Interestingly, PVAT was more strongly correlated with BMI than with WHR and should be considered when evaluating PVAT if ultrasound is not available. Perimesenteric fat is also associated with adiposity. Further investigation is required to assess the associations between PVAT of other named arteries with measures of adiposity in older adults and patients with cardiovascular disease (CVD). In addition, given the vascular dysfunction associated with excess PVAT, correlations with factors such as blood flow and pressure should be considered.

## Introduction

Cardiovascular disease (CVD) is a global health concern, and obesity stands out as one of the most significant risk factors [1]. Perivascular adipose tissue (PVAT) exhibits a complex and multifaceted role in cardiovascular health. While healthy PVAT contributes to vascular well-being by releasing beneficial factors that regulate blood pressure and inhibit inflammation, its function can be significantly altered in the context of obesity [2]. In such cases, PVAT can become dysfunctional, characterized by an increased secretion of pro-inflammatory molecules and alterations in cellular composition. This shift toward a pro-inflammatory phenotype contributes to a chronic state of inflammation and potentially elevates the risk of developing CVD [3,4]. Therefore, it is crucial to differentiate between the protective role of healthy PVAT and the detrimental effects of excess or dysfunctional PVAT, particularly in individuals with obesity or metabolic disorders, when considering its implications for cardiovascular health [5,6]. Some leading causes of obesity in young adults include diet, sedentary lifestyle, stress, lack of sleep, and substance/medication use. The relationship between PVAT of the SMA and obesity measures, such as body mass index (BMI) and WHR, remains unclear. According to the Global BMI Mortality Collaboration, obesity is defined as a BMI exceeding 30 kg/m^2^, a WHR of 0.85 for women, and a WHR of 0.9 for men. WHR can be used to assess central adiposity, which has been associated with metabolic syndrome. The two different fat distributions are the intrabdominal fat and the subcutaneous fat. The intrabdominal distribution is linked to an increased risk of CVD compared to subcutaneous fat distribution [7]. The superior mesenteric artery (SMA) was chosen for this study due to its anatomical and physiological relevance to visceral adipose tissue (VAT). As a major vessel supplying blood to a significant portion of the VAT depot, the SMA provides a strategic location to investigate the relationship between PVAT and measures of adiposity. The SMA's relatively superficial location and size also make it amenable to ultrasound imaging, allowing for reliable measurements of PVAT thickness.

This descriptive cross-sectional study aimed to investigate the correlation between PVAT of the SMA, VAT, BMI, and WHR in healthy adults aged 18-30, using ultrasound sonography. To the best of our knowledge, there are no published studies comparing the relationship between PVAT of the SMA and central measures of adiposity. Increased visceral fat, commonly observed in central obesity, is correlated with decreased adipocytokine function and increased proinflammatory factors, contributing to metabolic disorders such as type 2 diabetes, obesity, metabolic syndrome, and non-alcoholic fatty liver disease [8]. Moreover, PVAT has been implicated in the regulation of vascular tone through adipocytokines, a process that may be affected in obesity, and can lead to an increase in vascular resistance [2]. Therefore, exploring the relationship between PVAT, VAT, BMI, and WHR could provide essential insights into cardiovascular risk assessment [9]. 

This study was conducted in accordance with ethical guidelines and approved by Northumbria University. Participants provided informed consent by signing a waiver and agreeing to the collection of anthropometric and sonographic measurements by the researchers. 

## Materials and methods

This study had a cross-sectional design. Thirty-one participants (17M and 14F) aged 18-30 years (mean 21.5 years) from St. George’s University’s Keith B. Taylor Global Scholars Program gave written consent, which was approved by the Institutional Review Board (IRB) of Northumbria University (2023-5683-4929). The participants in this study completed a demographic questionnaire containing sex, age, and ethnicity, as well as disclosing any medical conditions and medications. Participants aged <18 and >30 years were excluded from the study. None of the participants were excluded based on medication use or any underlying medical conditions. Prior to anthropometric and sonographic measurements, participants were asked to fast for eight hours to minimize postprandial SMA diameter and maintain consistency in measures of central obesity [10].

**Figure 1:**
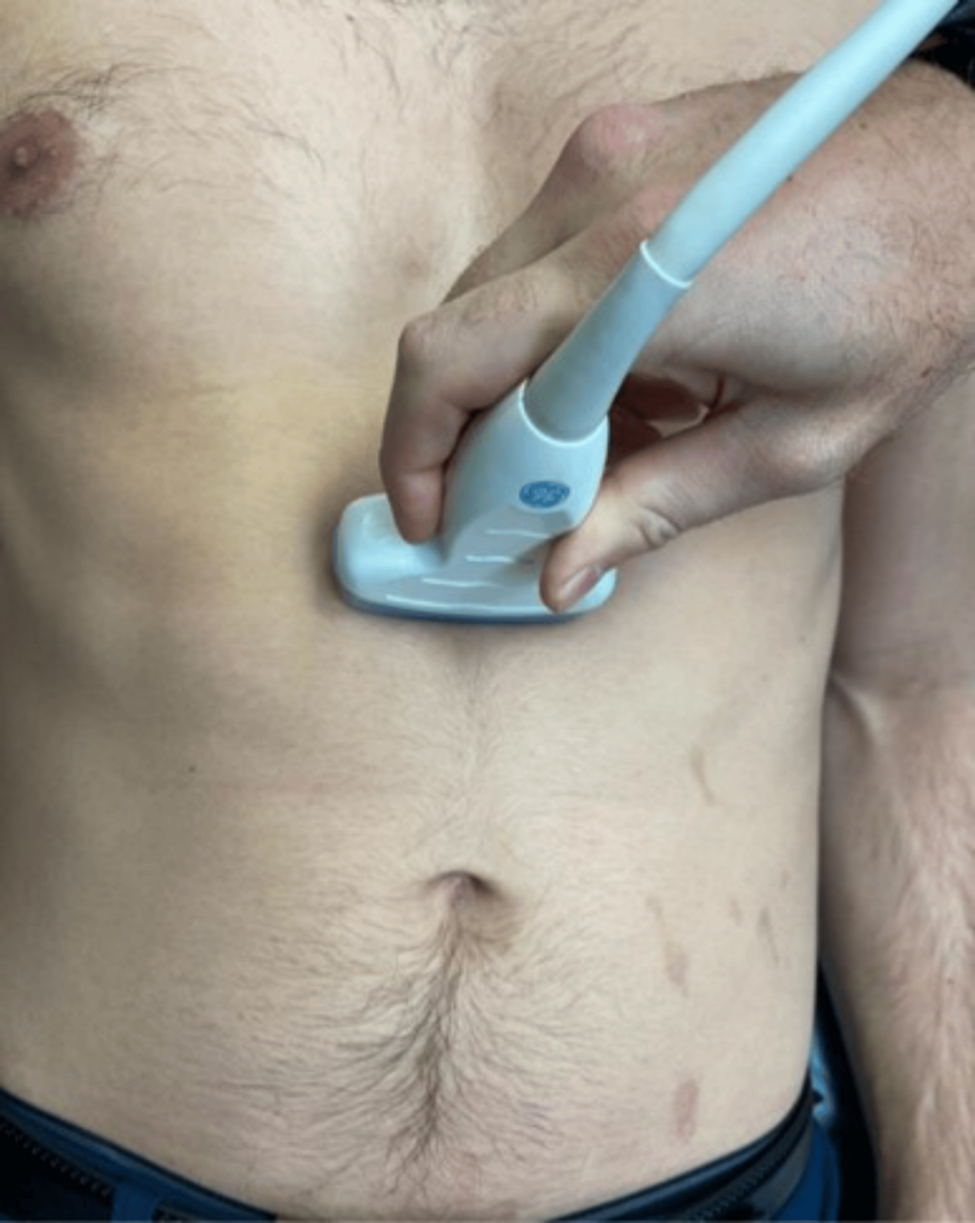
Horizontal Probe Placement for SMA and PVAT Measurement This figure illustrates the placement of the horizontal probe in the abdominal region, specifically 10 cm (approximately 3.94 in) above the umbilicus. The measurement was taken while the SMA was in a horizontal orientation. This position allowed for the precise measurement of PVAT surrounding the SMA. PVAT, perivascular adipose tissue; SMA, superior mesenteric artery

**Figure 2:**
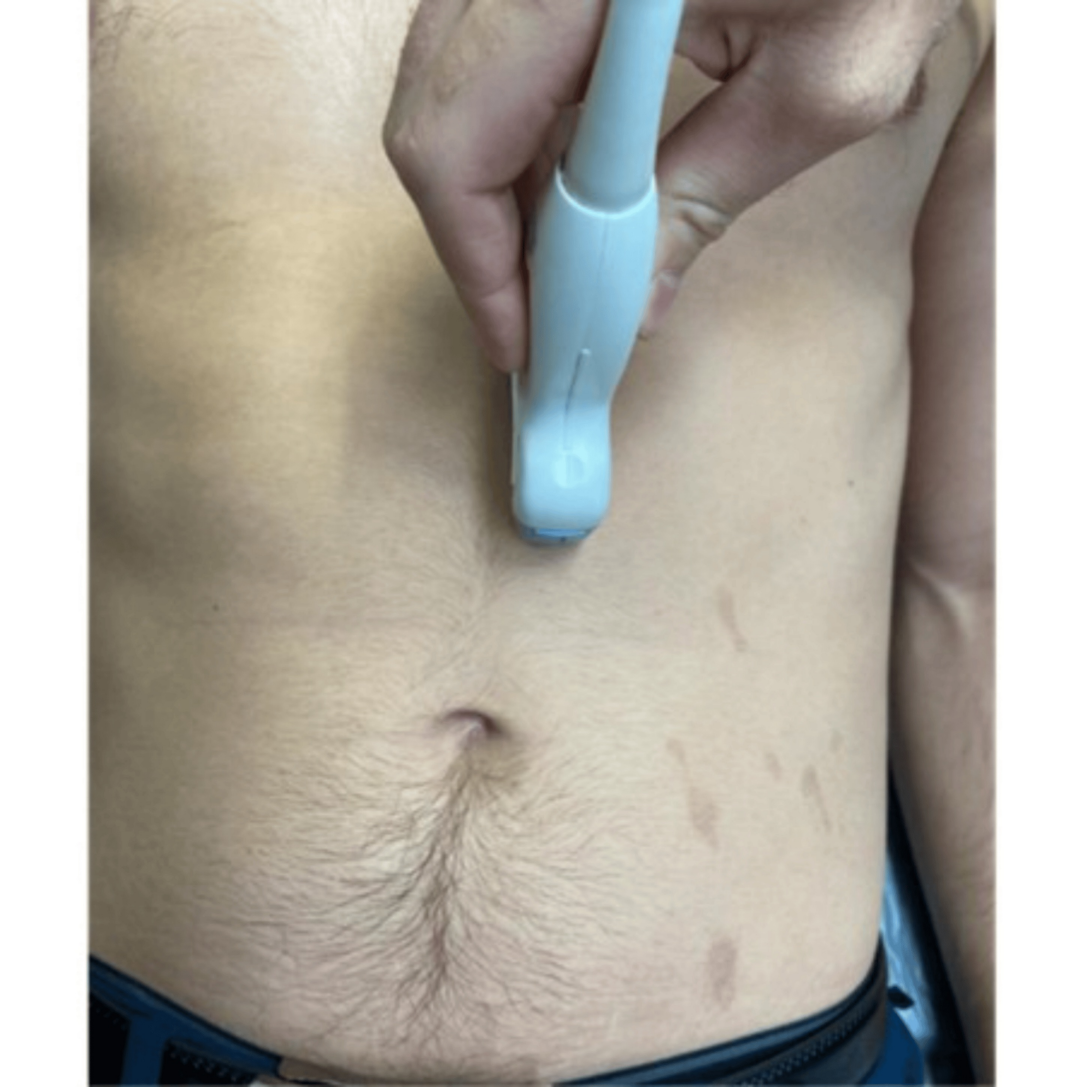
Probe Adjustment for Longitudinal Measurement of SMA and PVAT This figure demonstrates the adjustment of the probe to a longitudinal orientation in the abdominal region. The measurement was taken while the SMA was positioned longitudinally. This change in probe orientation allowed for a secondary measurement of PVAT surrounding the SMA. PVAT, perivascular adipose tissue; SMA, superior mesenteric artery

**Figure 3:**
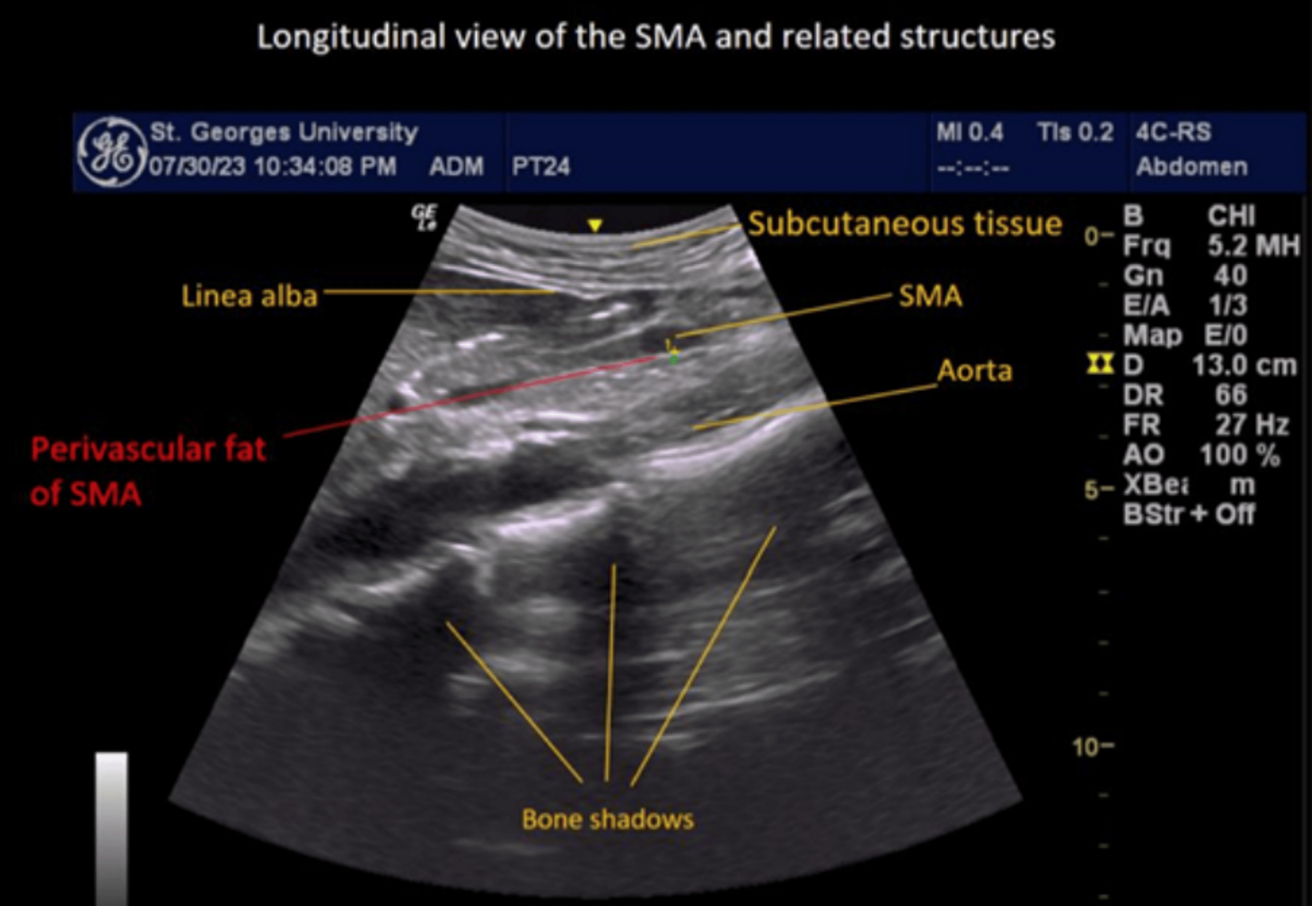
Ultrasound Image of the SMA and Surrounding Structures Ultrasound image of the SMA and its surrounding structures. The image highlights the subcutaneous tissue, linea alba, SMA, PVAT of the SMA, the aorta, and bone shadows. These components are clearly visible, providing a detailed depiction of the SMA and its associated PVAT. This image aids in understanding the SMA's anatomy and its relationship with nearby structures. SMA, superior mesenteric artery

**Figure 4:**
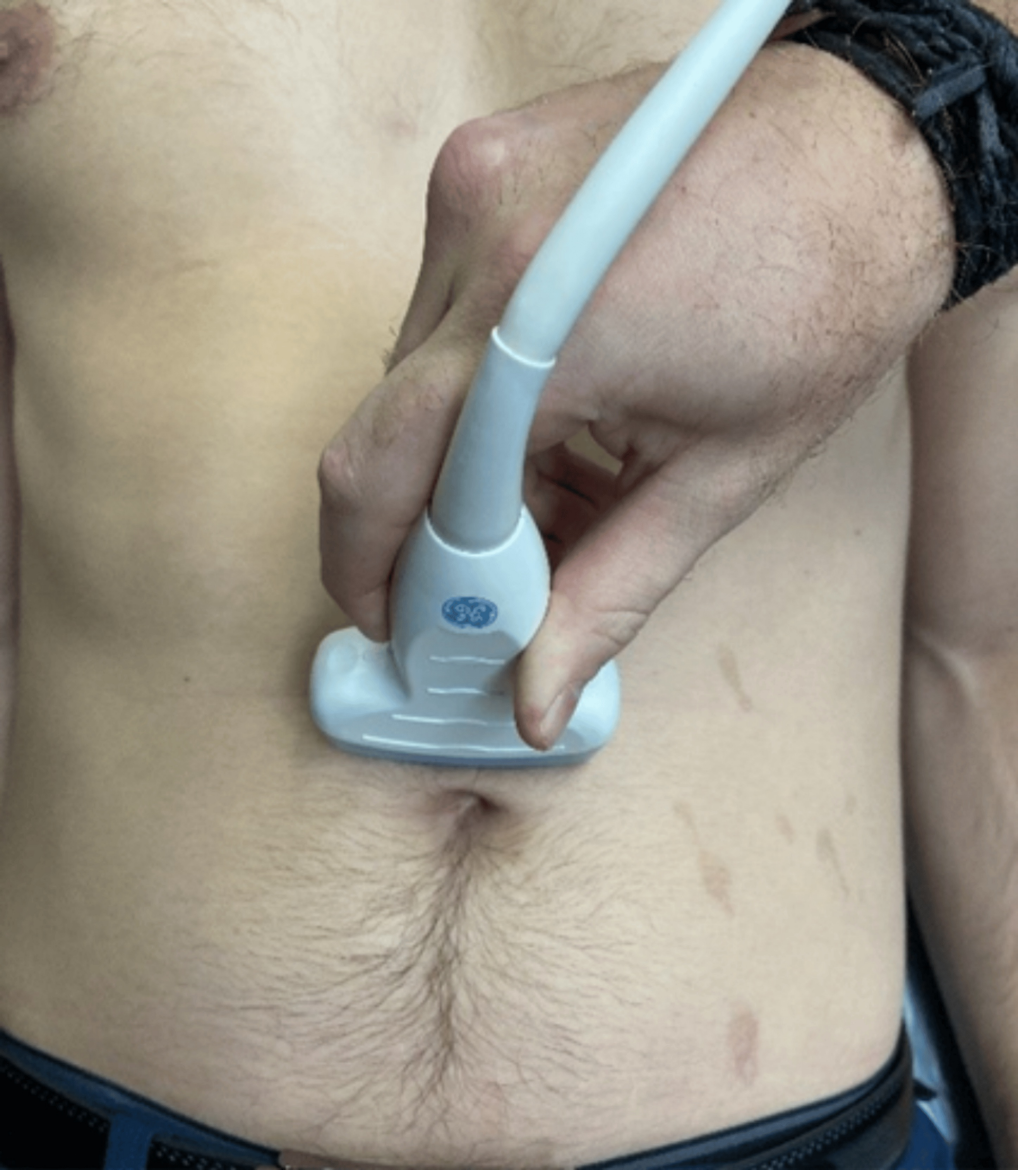
Probe Placement for VAT Measurement This figure demonstrates the placement of the horizontal probe in the abdominal area, positioned precisely 1 cm (approximately 0.39 in) above the umbilicus. This placement was chosen to measure VAT from the abdominal aorta to the Linea alba. The figure provides a visual representation of the technique used for VAT assessment in relation to anatomical landmarks. VAT, visceral adipose tissue

WHR and BMI were used as anthropometric measurements to assess the body composition. To minimize measurement error, the same researcher measured weight (in kg), height, waist, and hip measurements (in cm). Waist circumference was measured at the approximate midpoint between the lower margin of the last palpable rib, the top of the iliac crest, and the end of normal expiration. Hip circumference measurement was taken around the widest portion of the buttocks. Measurements were collected with stretch-resistant tape, snug around the body without constriction, and at a level parallel to the floor. BMI was measured by first having participants remove their shoes and step on the FITINDEX ES-26M-W scale. Height was measured in centimeters and subsequently converted to meters. BMI was then calculated using WHO’s standard equation: weight (kg)/m^2^). The GE LOGIQ ultrasound system with a 12 MHz abdominal probe transducer was used to identify the SMA transversely 10 cm (3.94 in) superior to the umbilicus. This was approximately at the level of L1/L2 vertebrae, where it was visible for all participants (Figure 1). While the probe was still in place, it was rotated 90° on the longitudinal axis (Figure 2), and the PVAT of the SMA was measured from the bottom of the SMA vessel (Figure 3). Finally, the probe was placed 1 cm (0.39 in) above the umbilicus transversely (Figure 4), and the VAT measurement was recorded, from the abdominal aorta to the Linea alba. Pearson’s correlation matrix was generated to assess the strength of the correlation between variables, and ANOVA was used to assess the statistical significance of Pearson’s correlation coefficient (r) values. 

## Results

Following the collection of measurements, data were analyzed using Microsoft® Excel. A Pearson’s correlation matrix was generated to assess the strength of the relationship between each of the variables of interest: sex, age, PVAT of the SMA, BMI, WHR, and VAT at the umbilicus. ANOVA was then used to assess the statistical significance of Pearson’s correlation coefficient (r) values. Differences were considered statistically significant at p<0.05. 

Thirty-one healthy adults aged 18 to 27 years (mean age: 21.5 years) participated in the study. Seventeen were male and 14 were female. The PVAT of the SMA was strongly and positively correlated with the VAT (r=0.77, p<0.001). Similarly, the PVAT of the SMA was moderately and positively correlated with BMI (r=0.61, p<0.001). In addition, a weakly positive but statistically significant relationship was measured between the PVAT of the SMA and WHR (r=0.38, p< 0.05) (See Table 1). 

**Table 1:** Measurement Variables Collected From 31 Participants, Average Values, and Standard Deviation WHR, waist-to-hip ratio

Measurement Variables	Average	Standard Deviation
Age (Years)	21.55	2.23
Height (m)	1.71	0.09
Weight (kg)	74.6	19.58
Basal Metabolic Rate	25.32	5.1
Waist Circumference (cm)	79.32	13.08
Hip Circumference (cm)	103.17	11.48
WHR	0.77	0.07

**Figure 5:**
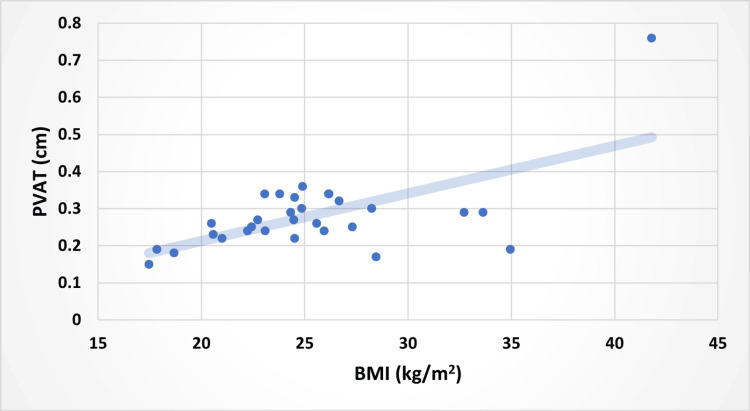
Relationship Between BMI and PVAT in the Participants With a Line of Best Fit PVAT, perivascular adipose tissue; BMI, body mass index

**Figure 6:**
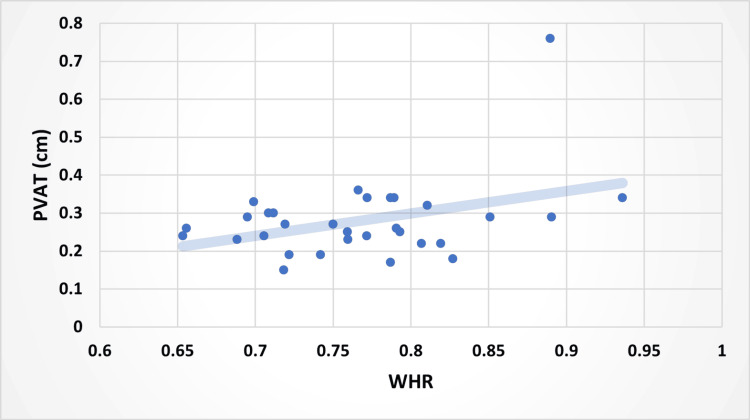
Relationship Between PVAT of SMA (cm) and WHR in the Participants With a Line of Best Fit PVAT, perivascular adipose tissue; SMA, superior mesenteric artery; WHR, waist-to-hip ratio

**Figure 7:**
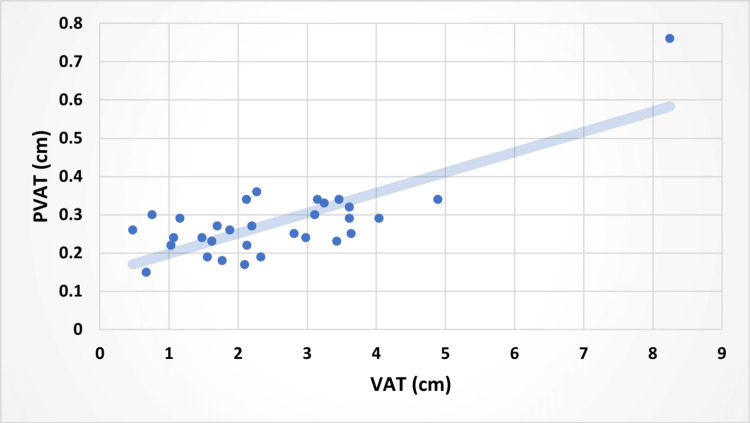
Relationship Between PVAT (cm) and VAT (cm) in the Participants With a Line of Best Fit PVAT, perivascular adipose tissue; VAT, visceral adipose tissue

Correlation between BMI and PVAT of the SMA (A), waist-to-hip ratio (WHR) and PVAT (B), and VAT vs. PVAT (C), as well as PVAT of the SMA (cm) and WHR (Figures 5, 6, 7).

## Discussion

Using ultrasound sonography, this study investigated the correlations between PVAT of the SMA, VAT, BMI, and WHR. The data demonstrated that PVAT was positively correlated with VAT, BMI, and WHR (Figures 5, 6, 7). Multiple studies have highlighted the role of PVAT in the development of atherosclerosis [11]. PVAT regulates many functions of the endothelial lining of the arteries. Specifically, the same study highlighted that at times of normal physiology, PVAT has been shown to have a protective effect on endothelial health via vasoactive molecules and anti-inflammatory cytokines [1]. However, PVAT can become dysfunctional under pathophysiological conditions such as metabolic syndrome [1,11]. Metabolic syndrome is a key factor in assessing health, as it increases the likelihood of serious issues, including stroke, peripheral artery disease, heart failure, type 2 diabetes, non-alcoholic fatty liver disease, kidney disease, sleep apnea, certain cancers, cognitive decline, and dementia [12].

In order to diagnose metabolic syndrome, three of the following five criteria are required: waist circumference exceeding 40 in for men (35 in for women), dyslipidemia, hypertriglyceridemia, high blood pressure, and impaired glucose levels [13,14]. Waist circumference is easily measured in clinical settings and approximates an individual's visceral fat [6,15]. Therefore, our study demonstrated that WHR can serve as a proxy for both visceral fat (C) and PVAT around the SMA in the absence of ultrasound measurement (Figure 6). The SMA was used to measure PVAT because it is a major artery embedded within the viscera and is easily identified on ultrasound, allowing for precise measurement of PVAT. Although excessive PVAT has been linked with signs of inflammation associated with the release of cytokines, a significant loss of the SMA fat pad can cause a reduction in the angle between the SMA and the abdominal aorta. An angle of less than 25 degrees or a reduction in the distance of less than 8 mm between the SMA and aorta can put an individual at risk for SMA syndrome. SMA syndrome is a rare disorder where the third part of the duodenum is compressed between the SMA and abdominal aorta. Loss of the SMA fat pad and compression of the duodenum can be caused by but not limited to rapid weight loss, cachexic states, surgical interventions, and congenital malformations [16].

Our study demonstrated an association between PVAT and VAT around the SMA, along with other markers of obesity, such as BMI, and, to a lesser extent, WHR, in young adults. Although significant, our study was limited in its findings because we did not measure any signs of metabolic dysfunction other than visceral adiposity. Therefore, this study did not provide any evidence to support the dysfunction or pathophysiology of PVAT in any of our participants. Thus, we cannot determine whether the correlation between the PVAT and VAT in young adults impacts PVAT dysfunction. 

Our study investigated both WHR and VAT as metrics of visceral adiposity, as these two variables both measure central adiposity and are expected to be relatively representative of each other [17,18]. Furthermore, WHR and VAT have been found to play significant roles in metabolic syndrome and should be considered when diagnosing metabolic syndrome [6,7,19]. Interestingly, PVAT was more strongly associated with VAT (r=0.77) and BMI (r=0.61) than was with WHR (r=0.36). Given that WHR is an approximate measure of VAT, a stronger correlation between PVAT and WHR was expected. A larger sample size and a more diverse patient population may have resulted in a different result.

The results were limited to young and healthy individuals. While the sexes were approximately well represented (17M and 14F), we had a limited representation of BMI classes. Nine participants were classified as overweight, four as obese, two as underweight, and 15 in the healthy weight range. Based on these demographic characteristics, our data is not externally valid for the general population. To truly assess the correlation between central measures of adiposity and PVAT of the SMA, a similar study design should include an older population. The results from future studies could be different from the results of our study since the prevalence of metabolic syndrome and CVD significantly increases with age [20,21]. In addition, our sample was not screened for pathological anomalies; therefore, we could not determine the metabolic state of the participants. However, further studies should be conducted to compare PVAT between people with and without metabolic syndrome. This would allow for a better understanding of the pathophysiology of PVAT with regard to its effects on metabolic syndrome. 

Future applications of ultrasound-mediated PVAT and VAT measurements can potentially aid doctors in finding an affordable and rapid way to assess cardiovascular risk in settings where ultrasound machines are readily available. Dysfunction of the PVAT is correlated with increased blood pressure in rodent models and plays an important role in vascular pathologies in vivo [22]. In humans, the anti-contractile effect of PVAT is lost in obese patients [14]. In an obese state, PVAT undergoes inflammation and hypoxia leading to a heightened expression of pro-inflammatory cytokines, increased reactive oxygen species, and thus a pathophysiological cascade that is detrimental to an obese individual [22,23]. More studies are needed to identify and categorize cardiovascular risk based on the radius of the PVAT in relation to the degree of pro-inflammatory cytokine release, in various BMI states. Therefore, with a standardized numerical PVAT level measurement and the resulting degree of proinflammatory cytokine function, one can potentially obtain an approximation of cardiovascular risk instead of performing invasive procedures such as lab tests and stress tests. 

Further research can be conducted to determine the negative correlation between PVAT and insulin sensitivity and how post-ischemic increases in blood flow affect vessels locally and systemically [24,25]. Studies have shown that PVAT-derived adipokines have both contractile and vasodilator functions, which can be dysfunctional in obesity [23,26,27]. More studies should be conducted to determine the effect of PVAT and arterial blood flow locally in various high-risk regions such as the coronary vessels and its correlation with obesity and insulin resistance. 

## Conclusions

PVAT is positively associated with multiple measures of adiposity and plays a crucial role in the development of CVD. However, PVAT as an individual risk factor for metabolic syndrome is yet to be clearly established. Our study found that PVAT of the SMA was more strongly correlated with VAT and BMI than WHR. Due to this unexpected correlation, further studies should assess the PVAT of the SMA in larger and older populations, as this may provide another valuable risk factor for CVD. In addition, the PVAT of other major arteries in relation to blood flow within the vessel could enhance our understanding of the physiology and pathophysiology of PVAT. Further research in this area can shed light on preventive and therapeutic measures to address obesity-related cardiovascular complications. 

## References

[1] Carbone S, Canada JM, Billingsley HE (2019). Obesity paradox in cardiovascular disease: where do we stand?. Vasc Health Risk Manag.

[2] Qi XY, Qu SL, Xiong WH, Rom O, Chang L, Jiang ZS (2018). Perivascular adipose tissue (PVAT) in atherosclerosis: a double-edged sword. Cardiovasc Diabetol.

[3] Kagota S, Maruyama-Fumoto K, Iwata S (2018). Perivascular adipose tissue-enhanced vasodilation in metabolic syndrome rats by Apelin and N-acetyl⁻l-cysteine-sensitive factor(s). Int J Mol Sci.

[4] Chen Y, Qin Z, Wang Y, Li X, Zheng Y, Liu Y (2021). Role of inflammation in vascular disease-related perivascular adipose tissue dysfunction. Front Endocrinol (Lausanne).

[5] Stanek A, Brożyna-Tkaczyk K, Myśliński W (2021). The role of obesity-induced perivascular adipose tissue (PVAT) dysfunction in vascular homeostasis. Nutrients.

[6] Carmienke S, Freitag MH, Pischon T, Schlattmann P, Fankhaenel T, Goebel H, Gensichen J (2013). General and abdominal obesity parameters and their combination in relation to mortality: a systematic review and meta-regression analysis. Eur J Clin Nutr.

[7] Fox CS, Massaro JM, Hoffmann U (2007). Abdominal visceral and subcutaneous adipose tissue compartments: association with metabolic risk factors in the Framingham Heart Study. Circulation.

[8] Landecho MF, Tuero C, Valentí V, Bilbao I, de la Higuera M, Frühbeck G (2019). Relevance of leptin and other adipokines in obesity-associated cardiovascular risk. Nutrients.

[9] (2023). CDC: Adult obesity facts. https://www.cdc.gov/obesity/data/adult.html.

[10] Moneta GL, Taylor DC, Helton WS (1988). Duplex ultrasound measurement of postprandial intestinal blood flow: effect of meal composition. Gastroenterology.

[11] Grigoras A, Amalinei C, Balan RA, Giusca SE, Caruntu ID (2019). Perivascular adipose tissue in cardiovascular diseases-an update. Anatol J Cardiol.

[12] Mathieu P, Pibarot P, Després JP (2006). Metabolic syndrome: the danger signal in atherosclerosis. Vasc Health Risk Manag.

[13] Dobrowolski P, Prejbisz A, Kuryłowicz A (2022). Metabolic syndrome - a new definition and management guidelines: A joint position paper by the Polish Society of Hypertension, Polish Society for the Treatment of Obesity, Polish Lipid Association, Polish Association for Study of Liver, Polish Society of Family Medicine, Polish Society of Lifestyle Medicine, Division of Prevention and Epidemiology Polish Cardiac Society, "Club 30" Polish Cardiac Society, and Division of Metabolic and Bariatric Surgery Society of Polish Surgeons. Arch Med Sci.

[14] Huang Cao ZF, Stoffel E, Cohen P (2017). Role of perivascular adipose tissue in vascular physiology and pathology. Hypertension.

[15] Bosomworth NJ (2019). Normal-weight central obesity: unique hazard of the toxic waist. Can Fam Physician.

[16] Singal R, Sahu PK, Goel M (2010). Superior mesenteric artery syndrome: a case report. N Am J Med Sci.

[17] Ashwell M, Cole TJ, Dixon AK (1985). Obesity: new insight into the anthropometric classification of fat distribution shown by computed tomography. Br Med J (Clin Res Ed).

[18] Onat A, Avci GS, Barlan MM, Uyarel H, Uzunlar B, Sansoy V (2004). Measures of abdominal obesity assessed for visceral adiposity and relation to coronary risk. Int J Obes Relat Metab Disord.

[19] Jabłonowska-Lietz B, Wrzosek M, Włodarczyk M, Nowicka G (2017). New indexes of body fat distribution, visceral adiposity index, body adiposity index, waist-to-height ratio, and metabolic disturbances in the obese. Kardiol Pol.

[20] Dominguez LJ, Barbagallo M (2016). The biology of the metabolic syndrome and aging. Curr Opin Clin Nutr Metab Care.

[21] Gouveia ÉR, Gouveia BR, Marques A (2021). Predictors of metabolic syndrome in adults and older adults from Amazonas, Brazil. Int J Environ Res Public Health.

[22] Aghamohammadzadeh R, Greenstein AS, Yadav R (2013). Effects of bariatric surgery on human small artery function: evidence for reduction in perivascular adipocyte inflammation, and the restoration of normal anticontractile activity despite persistent obesity. J Am Coll Cardiol.

[23] Huang PL (2009). A comprehensive definition for metabolic syndrome. Dis Model Mech.

[24] Lee HY, Després JP, Koh KK (2013). Perivascular adipose tissue in the pathogenesis of cardiovascular disease. Atherosclerosis.

[25] Britton KA, Fox CS (2011). Perivascular adipose tissue and vascular disease. Clin Lipidol.

[26] Lin A, Dey D, Wong DT, Nerlekar N (2019). Perivascular adipose tissue and coronary atherosclerosis: from biology to imaging phenotyping. Curr Atheroscler Rep.

[27] Greenstein AS, Khavandi K, Withers SB (2009). Local inflammation and hypoxia abolish the protective anticontractile properties of perivascular fat in obese patients. Circulation.

